# Sex Differences in Overall Survival and the Effect of Radiotherapy in Merkel Cell Carcinoma—A Retrospective Analysis of A Swedish Cohort

**DOI:** 10.3390/cancers13020265

**Published:** 2021-01-12

**Authors:** Hannah Björn Andtback, Viveca Björnhagen-Säfwenberg, Hao Shi, Weng-Onn Lui, Giuseppe V. Masucci, Lisa Villabona

**Affiliations:** 1 Department Oncology-Pathology, Karolinska Institute and BioClinicum, Karolinska University Hospital, 17176 Stockholm, Sweden; Hannah.bjorn-andtback@sll.se (H.B.A.); hao.shi@ki.se (H.S.); weng-onn.lui@ki.se (W.-O.L.); Giuseppe.masucci@ki.se (G.V.M.); 2 Department of Reconstructive Plastic Surgery, Karolinska University Hospital, 17176 Stockholm, Sweden; Viveca.bjornhagen-safwenberg@sll.se

**Keywords:** merkel cell carcinoma, merkel cell polyoma virus, sex, radiotherapy

## Abstract

**Simple Summary:**

Merkel cell carcinoma (MCC) is a rare and aggressive skin cancer which is believed to be partially caused by a virus or ultraviolet exposure. Most previous studies have shown that MCC is more common in men compared to women, virus associated MCC has a better prognosis and surgery followed by radiotherapy gives a better outcome. In this article, we explore these traits in a Swedish cohort of 113 patients and find that MCC is more common in women and female patients have a longer survival compared to male patients. In addition, we found that virus negative MCC has a worse outcome in male patients and radiotherapy after surgery gives a better outcome for patients who are treated with a curative dosage, irrespective of sex.

**Abstract:**

Merkel cell carcinoma (MCC) is a rare and aggressive skin cancer where Merkel cell Polyomavirus (MCPyV) contributes to the pathogenesis. In an adjuvant setting, radiotherapy (RT) is believed to give a survival benefit. The prognostic impact of sex related to MCPyV-status and adjuvant RT were analyzed in patients referred to Karolinska University Hospital. Data were collected from 113 patients’ hospital records and MCPyV analyses were made in 54 patients (48%). We found a significantly better overall survival (OS) for women compared to men and a significant difference in OS in patients receiving adjuvant RT. Furthermore, we found that men with virus negative MCC have an increased risk for earlier death (HR 3.6). This indicates that MCPyV positive and negative MCC act as two different diseases, and it might be due to different mechanism in the immune response between male and female patients. This could have significance in tailoring treatment and follow-up in MCC patients in the future.

## 1. Introduction

Merkel cell carcinoma (MCC) is a rare and highly malignant neuroendocrine skin cancer that mainly affects older people. The yearly incidence is 2500 in the United States and Europe and 60 cases in Sweden [1,2,3]. The disease mortality in MCC is as high as 46% within five years [4]. The rarity of the disease and its tendency to affect the elderly has contributed to MCC being little studied and the needs for novel prognostic and predictive biomarkers and new treatment regimens are substantial. Although rare, MCC has in several reports shown a rise in incidence over the last decades [2,5,6,7].

Merkel cell polyomavirus (MCPyV) was discovered in 2008, which was shown to be clonally integrated in the DNA of up to 80% of MCC tumors [8,9]. The presence of the virus has since been reported to be a favorable prognostic trait in MCC [10,11,12]. A trend towards women having a better outcome in MCC has been seen previously [13,14] and a recent finding from a large cohort in the U.S. establishes that women do have a better disease specific survival than men [15].

Several clinical risk factors for developing MCC have been identified, but much is still to be learned about the pathogenesis. Besides MCPyV, other risk factors are advanced age, chronic immunosuppression and prolonged ultraviolet (UV) exposure, therefore the primary tumor most often is found on sun exposed skin [16,17]. Curative treatment for MCC with localized disease consists of surgical resection of the primary tumor and the addition of postoperative (adjuvant) radiotherapy (RT) which in some settings has been shown to give a reduced risk of occurrence and survival advantage in a subgroup of patients [18]. However, a study of clinical outcomes and variables for a Swedish MCC-cohort has to our knowledge not yet been published.

In recent years, with the birth of immune checkpoint inhibitors, a new treatment option was born for patients with MCC [19,20]. Considering the pathogenesis of viral association and/or UV radiation, which is prone to cause a high tumor mutational burden [21], the immunogenicity of MCC should not be surprising. Furthermore, patients with a T cell dysfunction were shown to have an increased risk of developing MCC [22,23,24].

Our aim with this study was to analyze outcomes with regard to sex, adjuvant RT and MCPyV-status in a Swedish cohort, in order to improve the knowledge of MCC and identify prognostic traits for a better understanding of the possibility to tailor treatment and follow up strategies in the future.

## 2. Results

### 2.1. Cohort Characteristics and Overall Survival

**Figure 1:**
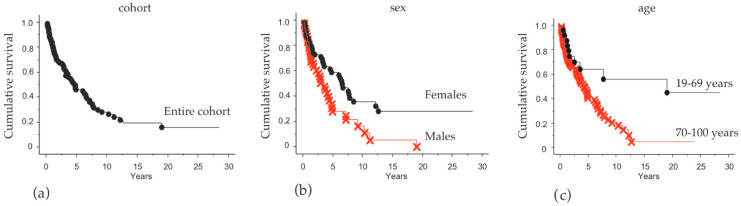
Overall survival analysis in relation to gender and age. Kaplan–Meier plots illustrating overall survival (OS) in: (**a**) the entire cohort; (**b**) Female (black) vs. Male (red) *p* = 0.04; and (**c**) age groups 19–69 years (black) vs. >70 years (red) *p* = 0.005.

In total, 113 patients, mostly living in the Stockholm Region and referred to Karolinska University Hospital between 1 January 1987 and 31 December 2019, diagnosed with MCC and treated with a curative intent, were included in the study. Detailed data on the patients are presented in Table 1. Of the patients, 64 were female (57%) and 49 were male (43%). Median age at surgery was 76 years (range 19–100) for the entire cohort, for women 79 years (range 19–100) and for men 75 years (range 59–94). There was a clear difference in overall survival (OS) between patients aged 19–69 years (younger: 22%) and >70 years (older: 78%) (Figure 1).

The localization of the primary tumor was distributed with a large proportion in the head and neck region (47%) and the others divided among upper extremity (21%), lower extremity (18%), trunk (11%) and genital area (3%) (Table 1).

The patients presented with clinical stages I–III and the majority was stage I (57%) followed by stage II (31%) and stage III (12%).

At the end of the observation period, the probability of survival in the entire cohort was 16% (Figure 1a). There was significantly higher OS for women (30%) compared to men (*p* = 0.04; Figure 1b). Patients under 70 years old had a better outcome (46%) than older patients (*p* = 0.005; Figure 1c). There was no statistically significant difference in outcome between clinical stages.

Patients who had the primary MCC localized in the extremities had a better outcome compared to other anatomical sites (Table 2). This was seen both in entire cohort (HR 0.48) and in the female patients (HR 0.35) for extremities vs. trunk. The comparison of extremities vs. head and neck region was statistically significant when comparing the whole cohort, but only a tendency when separated by sex. There was no significant difference between localization in the head and neck region compared to the trunk (Table 2).

### 2.2. MCPyV-Status and Overall Survival

**Figure 2:**
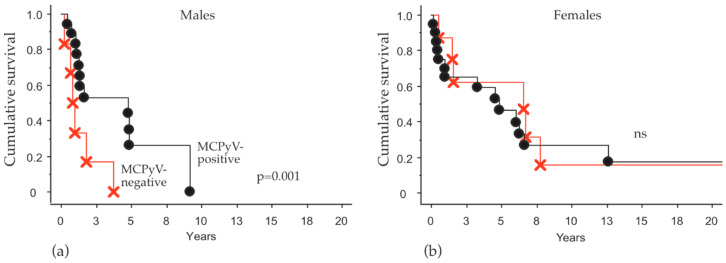
Overall survival analysis in male patients in relation to MCPyV status. Kaplan–Meier plots illustrating overall survival (OS) in (**a**) male and in (**b**) female patients with MCPyV-positive (black) or MCPyV-negative (red) tumor tissue, *p* = 0.001.

Tumor samples from 54 patients (47%) were available for the detection of MCPyV in tumor tissue (Table 1). In these samples, 74% were positive and 26% negative. The distribution by sex was similar: 72% positive and 28% negative in the female patients and 76% positive and 24% negative in the male patients. A comparison between male and female patients for the risk to die due to MCPyV status is shown in Table 3. Among the 54 MCC patients with MCPyV status, there was no difference in the risk for negative or positive patients. However, male patients with virus-negative MCC had an increased risk for death compared to male patients with virus-positive tumors (HR 3.6; 95% CI, 1.2–10; *p* = 0.018). Using Kaplan–Meier survival analysis, a better survival was also observed in the MCPyV positive male patients (Figure 2). Female patients’ viral status had no impact on OS in this analysis (Table 3).

### 2.3. Treatment and Overall Survival

In this cohort, 66 (58%) patients were treated with surgery alone and 47 (42%) patients received radiotherapy in a variety of regimens (Table 1).

**Figure 3:**
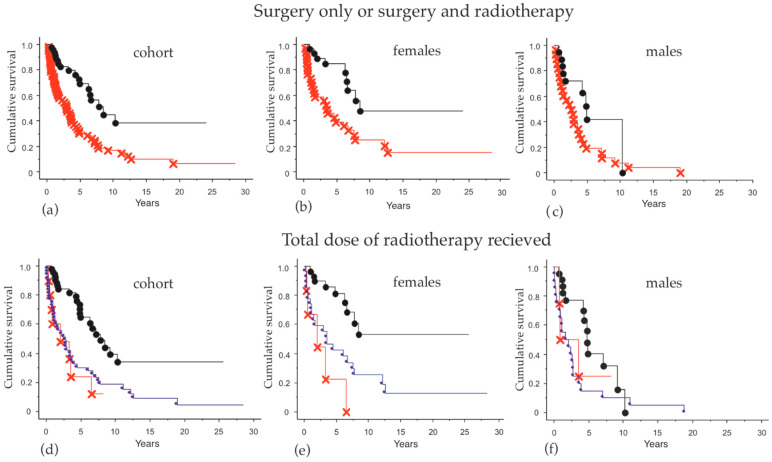
Overall survival in patients receiving surgery alone, a combination of surgery and adjuvant radiotherapy (RT), as well as radiation doses (**a**–**c**). Comparisons of overall survival between patients treated with surgery alone (red) and surgery plus adjuvant RT (black) in: (**a**) the entire cohort, *p* = 0.0001; (**b**) female patients only, *p* = 0.002; and (**c**) male patients only, *p* = 0.03. (**d**–**f**) Comparisons of overall survival among patients treated with radiation ≥50 Gy (black), <50 Gy (red) and patients never exposed to radiotherapy (blue) in: (**d**) the entire cohort, *p* = 0.0001; (**e**) only female, *p* = 0.0005; and (**f**) only male patients, *p* = 0.07.

Patients who received adjuvant RT after surgery had a significant benefit for survival (*p* = 0.0001) (Figure 3a). No difference was detected between male and female patients (Figure 3b,c).

In addition, we analyzed the efficacy of radiotherapy in cases where relapse was detected. For this reason, the patients were divided into three subgroups considering the total amount of radiation (palliative and adjuvant) received during their disease process (never exposed to RT, RT < 50 Gy and RT ≥ 50 Gy) (Figure 3d–f).

Patients who received ≥50 Gy had a better outcome compared to patients who received a lower dose. The latter group did not differ from patients who never received radiation and this tendency was most explicit in the female group of patients.

**Figure 4:**
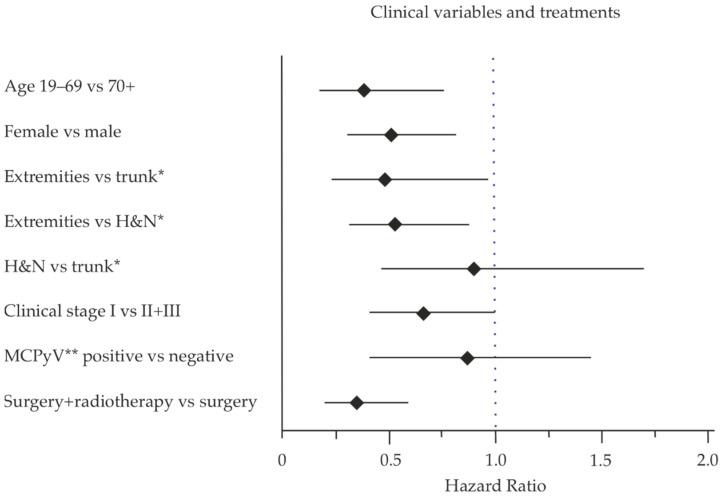
Forest plot for the hazard ratio of the clinical variables and MCPyV-status. * For both sexes, differences are shown in Table 2. ** For both sexes, differences are shown in Table 3.

Univariate and multivariate analysis of the risk (Cox–Mantel) was performed on the clinical variable collected and summarized in a forest plot (Figure 4). The figure summarizes the findings of the prognostic variables investigated, where younger age, tumor location on extremity and radiotherapy treatment were associated with a better outcome, while male sex was a factor for a worse outcome and increased risk for death.

## 3. Discussion

In this study, we reviewed clinical data and outcomes for 113 MCC patients from the Stockholm region who were referred to the Karolinska University Hospital in Stockholm, Sweden. The rarity of MCC makes the relatively small number a large cohort by Nordic standards and to our knowledge the largest historical cohort with clinical outcomes described in Sweden. We utilized data from patient hospital records and cause of death registry, as well as MCPyV status available in tumor tissue. The data were correlated to overall survival and sex in addition to treatment received.

In our cohort, we show a better overall survival in patients receiving adjuvant RT after surgery compared to patients who were treated with surgery alone.

Adjuvant RT for MCC has been used in selected cases since the 1970s at Karolinska University Hospital; however, it was only since the late 1980s that a definite treatment schedule has been applied for adjuvant purposes. Very little is presented in the literature in this respect. Despite all the limitations, most retrospective analyses show with relatively clear consensus that adjuvant RT reduces recurrence [25,26,27], and only two other studies have shown a positive impact on overall survival [28,29]. Both studies are large retrospective MCC cohorts investigating the benefit of adjuvant RT. Chen et al. analyzed 4815 patients with MCC in the head and neck region and showed a survival benefit from adjuvant RT in patients with narrow surgery margins, large tumors and male sex [28]. Bhatia et al. analyzed 6908 patients and reported a benefit both for local recurrence and overall survival in patients with stage I and II disease, but not stage III [29]. Our results show that patients receiving radiotherapy had a clear survival benefit compared to patients who received surgery alone. In our much smaller cohort, we clearly see a survival benefit in both male and female patients receiving adjuvant RT > 50 Gy. Patients who received <50 Gy were most likely offered radiotherapy with palliative intent, which may be the reason for their much worse prognosis. Even though the number of patients was insufficient to analyze any benefit for patients in different clinical stages, our results strengthen the international consensus that MCC patients should be offered adjuvant RT.

Our findings also show that female patients, regardless of MCPyV status, had a significantly improved OS compared to male patients. This finding is also supported by a recent report from an analysis of a large cohort of MCC cases in the US [15].

Previous analysis has shown an inconsistency of the prognostic traits of MCPyV; some studies have shown that patients with MCPyV-positive tumors have a more favorable outcome, whereas others have either found it to be unclear or even prognostically unfavorable [10,11,12,30,31,32,33]. MCPyV-positivity and better outcome was a trend in our material, but the results were non-significant. Interestingly, when we made a multivariate analysis with sex and MCPyV-status, we found that the male patients with MCPyV-negative tumors had the worst outcome and a significantly higher risk for death compared to male patients with MCPyV positive tumors (HR 3.6). The MCPyV status of female patients did not affect outcome in our cohort. This novel finding may serve as a prognostic marker, where male patients and especially virus negative ones, could benefit from closer clinical monitoring and evaluation after primary treatment.

The differences in MCPyV-positive and negative MCC have been extensively researched [34], some even going as far as suggesting that MCPyV-negative MCC does not exist [35]. Our findings in gender differences in outcome may add another dimension to previous findings.

Considering the immunogenicity of MCC, however, one may raise the question of whether these differences in outcome of the patients regarding sex could be due to different immune responses between men and women. Several publications [36,37,38,39] have explored both the difference in outcome of immunotherapy treatment between men and women, but also the differences in immune response between the sexes [38]. Given the immunogenicity of MCC, additional studies of immunological markers, such as CD8+ lymphocyte infiltration, MHC class I expression and HLA-genotype would be of interest to further shed light on the sex differences in the immune response. The novel treatment options of immunotherapy for MCC and the reports of the differences in immune response between male and female indicates that sex may play a role in the future treatment options for these patients.

Male sex has been described as an independent risk factor for developing MCC [1,2]. However, in our cohort, we found a shift towards female patients (64%). Similar results have recently been reported from a Finnish study where female patients constituted 65% [40]. The increased incidence in female patients in a Swedish cohort was also previously discussed by Zaar et al. [6] who calculated the age adjusted incidence as higher in male patients. This finding may suggest that there are differences in the sex distribution in the older populations between the Nordic countries compared to the cohorts previously described. It does not, however, explain the differences in outcome between male and female patients discussed above.

Another clinical parameter that had an impact on OS was age, which unsurprisingly showed a better OS in younger patients (19–69) compared to older patients (>70). The distribution of men and women in these groups was somewhat uneven, however the impact on our findings should be limited considering the median age was higher for the female group compared to the male group (79 and 76 years, respectively). Clinical stage could also have an impact on OS; however, these groups were evenly distributed between the sexes (Table 1). Clinical stage in itself did not show a statistically significant difference in OS (not shown), however this may be due to the limited number of stage III patients (*n* = 14, Table 1) who all received adjuvant RT which may have a positive impact on their outcome.

The most common anatomical location for the primary tumor was in the head and neck region (47%) and the next most common anatomical location was the upper extremities (21%), which are consistent with other publications and no difference between men and women [14,34,41].

We acknowledge several limitations of this historic cohort analysis. The main limitation is the sample size due to the rare nature of MCC, however these findings still add insight to several important prognostic traits in curative patients with MCC.

## 4. Materials and Methods

### 4.1. Patient Selection

Patients diagnosed with MCC and referred to the plastic surgery unit at Karolinska University Hospital from 1987 until the end of 2019 were included in the study. Patients underwent primary surgery alone with additional scar excision and with wide margins or were assessed for adjuvant RT at the Onco-Radiation Therapy department of the hospital. Start date was set to the day of surgery. Censor date was set to death date or end-date of the study, 31 December 2019.

The pathology evaluation and diagnosis were mainly performed or reviewed by pathologists at Karolinska University Hospital at the time of diagnosis.

Survival data and given treatment were retrieved from patient hospital records, pathology reports, population registry and the Swedish cause of death registry. Largest diameter of the primary tumor was identified in patient records prior to surgery or from pathology reports. Tumor stage was assessed according to the 8th edition consensus staging system by the American Joint committee on Cancer (AJCC) published in 2017.

The study was conducted in accordance with ethical approval Dnr 2019-05951 approved by the Ethics Review Board (Etikprövningsnämnden) in Sweden.

### 4.2. Surgery

Patients with MCC stage I and II underwent radical tumor excision, preferably of 1–2 cm in margin down to muscle fascia, pericondrium or periosteum. The aim of surgery is to achieve free margins.

### 4.3. Radiotherapy Treatment

Radiotherapy as a treatment option for MCC has been a tradition at the Oncology and Radiotherapy department at Karolinska since the 1970s and post-operative radiotherapy has been widely used. Established practice is to offer 2 Gy per fraction, 5 fractions per week up to a total dose of 50 Gy or more. Bolus is used in selected cases to achieve adequate doses in the skin. Common margins have been 1–3 cm. For patients with microscopically or macroscopically positive margins a total dose of 56–66 Gy have been given. When radiotherapy treatment is given after relapse, doses vary depending on indication.

### 4.4. McPyV Analysis

MCPyV analyses were made by MCPyV LT immunohistochemistry using CM2B4 (Santa Cruz Biotechnology, Dallas, TX) or Ab3 (gift from Dr. J.A. DeCaprio) antibody and PCR detection of MCPyV DNA in tumor samples, as previously described [42]. The virus status of 40 patients was characterized in previous studies [42,43,44] and 14 patients were characterized in this study.

### 4.5. Statistical Analysis

Descriptive statistics for nominal or numeric variables was applied. When required distribution differences and correlations between categorical data were compared with the χ² test and ordinal data with the Spearman Rank’s test. This was used to examine relationships between patient’s demographics, clinical variables and biomarkers. Student *t*-test was used to compare mean values. Survival analysis was performed using the Kaplan–Meier method and differences in survival were tested with the log-rank test. Cox–Mantel regression was used in the univariate and multivariate analyses. The results were considered significant if *p* ≤ 0.05. Calculations were performed with the program StatView™ for Windows, SAS Institute Inc. Version 5.0.1. The Forest Plot presentation was performed using MedCalc™ program version 19.1.

## 5. Conclusions

Our data confirm the positive impact of RT on survival in a Swedish MCC cohort. Our findings also show not only that women have a better prognosis, but also that men with virus negative MCC have the worst outcome. Our findings thus indicate that MCPyV positive and negative MCC act as two different diseases and raise questions of whether there is a difference in the disease itself or the immune response towards MCC in male and female patients.

## Figures and Tables

**Table 1:** Patients clinical characteristics and treatments.

Cohort Characteristics		Cohort		Female		Male	
		n	%	n	%	n	%
Cohort		113	100	64	57	49	43
Age	Median, years	76		79		75	
	19–69	25	22	18	28	7	14
	>70	88	78	46	46	42	86
Tumor Location	Head and neck	53	47	30	47	23	47
	Upper extremity	24	21	14	22	10	20
	Lower extremity	20	18	13	20	7	14
	Trunk	12	11	5	8	7	14
	Genital area	4	4	2	3	2	4
Stage	I	64	57	36	56	28	57
	II	35	31	22	34	13	27
	III	14	12	6	9	8	16
MCPyV-Status in Tumor		54		29		25	
	Positive	40	74	21	72	19	76
	Negative	14	26	8	28	6	24
Treatment	Surgery	66	58	36	56	30	61
	Surgery and radiotherapy	47	42	28	44	19	39

**Table 2:** Overall survival comparison between primary tumor site.

Parameters	Extremities vs. Trunk			Extremities vs. H&N			H&N vs. Trunk		
	Cohort	Females	Males	Cohort	Females	Males	Cohort	Females	Males
Hazard	0.48	0.35	0.88	0.53	0.52	0.48	0.9	0.65	1.6
C.I. 95%	0.23–0.97	0.12–1.02	0.31–2.4	0.32–0.87	0.24–1.11	0.21–1.08	0.47–1.7	0.24–1.7	0.65–4.3
P	0.03	0.05	ns	0.034	ns	ns	ns	ns	ns

HR, Hazard ratio; CI, confidence interval; ns, not significant.

**Table 3:** Hazard ratios by Cox–Mantel regression analysis comparing MCPyV negative vs. positive filtered by sex.

Sample	MCPyV Negative vs. Positive		
	HR	95% C.I.	*p*-Value
MCPyV cohort (*n* = 54)	1.3	0.65–2.6	ns
Females (*n* = 29)	0.84	0.32–2.2	ns
Males (*n* = 25)	3.6	1.2–10	0.018

HR, hazard ratio; CI, confidence interval; ns, not significant.

## Data Availability

The data presented in this study are available on request from the corresponding author. The data are not publicly available due to them containing information that could compromise research participant privacy.
